# Predicting interval and screen-detected breast cancers from mammographic density defined by different brightness thresholds

**DOI:** 10.1186/s13058-018-1081-0

**Published:** 2018-12-13

**Authors:** Tuong L. Nguyen, Ye K. Aung, Shuai Li, Nhut Ho Trinh, Christopher F. Evans, Laura Baglietto, Kavitha Krishnan, Gillian S. Dite, Jennifer Stone, Dallas R. English, Yun-Mi Song, Joohon Sung, Mark A. Jenkins, Melissa C. Southey, Graham G. Giles, John L. Hopper

**Affiliations:** 1 Centre for Epidemiology and Biostatistics, The University of Melbourne, Level 3/207 Bouverie Street, Carlton, VIC 3053 Australia; 2 Cancer Epidemiology and Intelligence Division, Cancer Council Victoria, Melbourne, VIC Australia; 3 Curtin UWA Centre for Genetic Origins of Health and Disease, Curtin University and the University of Western Australia, Perth, Western WA 6009 Australia; 4 Department of Family Medicine, Samsung Medical Center, Sungkyunkwan University School of Medicine, Irwon-ro 81, Gangnamgu, Seoul 06351 South Korea; 5 Department of Epidemiology School of Public Health, Seoul National University, 1 Gwanak-ro, Gwanak-gu, Seoul, 151-742 Korea; 6 Department of Pathology, University of Melbourne, Carlton, Victoria 3053 Australia; 7 Institute of Health and Environment, Seoul National University, 1 Gwanak-ro, Gwanak-gu, Seoul, 151-742 Korea; 8 Department of Clinical and Experimental Medicine, University of Pisa, ᅟPisa, Italy; 9 Precision Medicine, School of Clinical Sciences at Monash Health, Monash University, Clayton, Victoria Australia

**Keywords:** Breast cancer, Masking effect, Interval cancer, Screen-detected, Nested case–control cohort study, Australian women, Mammography, Mammographic density

## Abstract

**Background:**

Case–control studies show that mammographic density is a better risk factor when defined at higher than conventional pixel-brightness thresholds. We asked if this applied to interval and/or screen-detected cancers.

**Method:**

We conducted a nested case–control study within the prospective Melbourne Collaborative Cohort Study including 168 women with interval and 422 with screen-detected breast cancers, and 498 and 1197 matched controls, respectively. We measured absolute and percent mammographic density using the Cumulus software at the conventional threshold (*Cumulus*) and two increasingly higher thresholds (*Altocumulus* and *Cirrocumulus*, respectively). Measures were transformed and adjusted for age and body mass index (BMI). Using conditional logistic regression and adjusting for BMI by age at mammogram, we estimated risk discrimination by the odds ratio per adjusted standard deviation (OPERA), calculated the area under the receiver operating characteristic curve (AUC) and compared nested models using the likelihood ratio criterion and models with the same number of parameters using the difference in Bayesian information criterion (ΔBIC).

**Results:**

For interval cancer, there was very strong evidence that the association was best predicted by *Cumulus* as a percentage (OPERA = 2.33 (95% confidence interval (CI) 1.85–2.92); all ΔBIC > 14), and the association with BMI was independent of age at mammogram. After adjusting for percent *Cumulus*, no other measure was associated with risk (all *P* > 0.1). For screen-detected cancer, however, the associations were strongest for the absolute and percent *Cirrocumulus* measures (all ΔBIC > 6), and after adjusting for *Cirrocumulus*, no other measure was associated with risk (all *P* > 0.07).

**Conclusion:**

The amount of brighter areas is the best mammogram-based measure of screen-detected breast cancer risk, while the percentage of the breast covered by white or bright areas is the best mammogram-based measure of interval breast cancer risk, irrespective of BMI. Therefore, there are different features of mammographic images that give clinically important information about different outcomes.

**Electronic supplementary material:**

The online version of this article (10.1186/s13058-018-1081-0) contains supplementary material, which is available to authorized users.

## Background

Mammographic images can be used for more than identifying existing breast tumours – they contain information that predicts both (1) the risk of future breast cancers and (2) the likelihood of existing tumours being missed due to masking [1]. This has important implications for future breast cancer control because of the widespread use of mammography screening. Women could be triaged according to their risk of disease and/or masking so as to implement tailored screening, and deal with the ongoing need to improve cost-effectiveness, minimise harm [2, 3], and help address the issue of “dense breasts” [4].

Conventionally, mammographic density has been defined as the white or bright areas on a mammogram and, after adjusting for age and body mass index (BMI), has been found to be a risk factor for both interval and screen-detected breast cancers [1, 5, 6]. Screen-detected breast cancers are cancers detected via a screening programme and interval breast cancers are those detected within 2 years of a mammographic screen that did not detect any breast cancer. Interval cancers are in general more aggressive [6–11], so it is important to understand if the relationship between mammographic density measures and breast cancer risk differs by mode of detection [12].

We previously found, from case-control studies of both Australian [13] and Korean [14, 15] women, that by defining mammographic density at higher than conventional pixel-brightness thresholds we obtained better discrimination between women with and without breast cancer. This applied for both film and digital mammograms. It is not known, however, if this observation differs according to mode of detection. In this study, we have used a prospective cohort and asked if, and how well, the conventional and our new mammographic density measures apply to interval and screen-detected breast cancers.

## Materials and methods

### Subjects

We used the prospective Melbourne Collaborative Cohort Study of Australian-born, Italian-born, and Greek-born male and female adult residents of Melbourne [16, 17]. We conducted a nested case–control study in which cases were women who had been diagnosed with a first diagnosis of ductal carcinoma in situ or invasive adenocarcinoma of the breast (C50.0-C50.9) since the baseline survey. Cases were ascertained by record linkage to the population-complete Victorian Cancer Registry and to the Australian Cancer Database. Controls had been matched for year at birth, year of baseline interview, and country of origin [12, 18]. Controls were selected randomly from those who had not been diagnosed with breast cancer at the age of diagnosis of the case. Participants completed interviewer-administered questionnaires that captured standard risk factors for breast cancer, including reproductive history. Height and weight were measured directly according to standard protocols and BMI was calculated from these. We studied only those female cohort members who had attended BreastScreen Victoria (the state-organised, free, 2-yearly, mammographic screening service for women aged 50 to 69 years) at least once for a mammogram, and did not have a diagnosis of breast cancer when they completed their baseline questionnaire. Family history of breast cancer in any blood relative, as collected by BreastScreen Victoria, was available for all participants.

Breast cancers were identified by BreastScreen Victoria, or by linkage with the Victorian Cancer Registry. Breast cancers diagnosed outside the screening service before the next scheduled scan (2 years) after a negative scan were classified as interval cancers, and those subsequently identified by BreastScreen Victoria at a scheduled scan were classified as screen-detected. Consequently, the study included two subgroups: 422 women with screen-detected breast cancers (357 of which were invasive) with 1197 matched controls and 168 women with interval breast cancers (148 of which were invasive) with 498 matched controls. For this study, we selected the mammogram closest to baseline (average 2 years difference [18]), and this was on average 5 or 6 years prior to diagnosis for interval and screen-detected cases, respectively; see Table 1 in a previous publication [12]. The study was approved by the human research ethics committees of the University of Melbourne and the Cancer Council Victoria.

**Table 1:** Characteristics (mean (SD), or number (percentage)) of patients (cases) and controls, by mode of detection (screen-detected or interval breast cancer)

	Interval cancer			Screen-detected cancer		
	Cases (*n* = 168) Mean (SD)	Controls (*n* = 498) Mean (SD)	*P* ^a^	Cases (*n* = 422) Mean (SD)	Controls (*n* = 1197) Mean (SD)	*P* ^a^
Age at mammogram (years)	56.0 (6.8)	56.2 (6.8)	0.2	58.8 (7.5)	58.4 (7.6)	0.007
Body mass index (kg/m^2^)	26.7 (5.3)	26.5 (4.9)	0.8	27.3 (5.0)	26.5 (4.8)	0.002
Number of live births	2.6 (0.9)	2.7 (0.9)	0.2	2.8 (1.0)	2.7 (0.9)	0.96
	*N* (%)	*N* (%)	*P*	*N* (%)	*N* (%)	*P*
Parity						
Yes	139 (83)	425 (85)	0.4	359 (85)	1046 (87)	0.3
No	29 (17)	73 (15)		63 (15)	151 (13)	
Menopausal status						
Premenopausal	67 (40)	197 (40)	0.6	127 (30)	359 (30)	0.3
Postmenopausal	101 (60)	301 (60)		295 (70)	838 (70)	
Breast feeding						
Yes	123 (73)	380 (76)	0.5	327 (77)	937 (78)	0.7
No	41 (25)	110 (22)		88 (21)	240 (20)	
Missing	4 (2)	8 (2)		7 (2)	20 (2)	
Family history of breast cancer						
Yes	44 (26)	75 (15)	0.001	91 (22)	165 (14)	< 0.001
No	124 (74)	423 (85)		331 (78)	1032 (86)	
Ever alcohol consumption						
Yes	119 (71)	318 (64)	0.08	248 (59)	759 (63)	0.2
Never	49 (29)	180 (36)		174 (41)	438 (37)	
Ever use of oral contraceptive						
Yes	102 (61)	324 (65)	0.2	247 (59)	711 (59)	0.8
Never	66 (39)	172 (35)		174 (41)	492 (40)	
Missing	0 (0)	2 (0)		1 (0)	4 (1)	
Ever use of hormonal therapy						
Yes	54 (32)	141 (28)	0.3	134 (32)	333 (28)	0.1
Never	113 (67)	355 (71)		284 (67)	859 (72)	
Missing	1 (1)	2 (1)		4 (1)	5 (0)	

*SD* standard deviation

^a^*P* indicates the significance of differences between cases and matched controls based on conditional logistic regression

### Measurement of mammographic density

The cases’ contralateral mammograms that had been taken at or before diagnosis were used for analysis. All mammograms were screen-film and were digitised by the Australian Mammographic Density Research Facility using an Array 2905 Laser Film Digitizer at 12-bit depth.

The Cumulus computer-assisted thresholding method was used to measure mammographic density [19]. Five of the authors (TLN, YKA, SL, CFE, and NHT) independently measured mammographic density blinded to case-control status, mode of detection, and any other density measures already performed on the mammogram. Mammographic density was measured using the conventional definition of the white or bright areas, and we call these measures *Cumulus*. Mammographic density was further defined as the bright or brighter areas, in effect at higher pixel-brightness thresholds, and we call these measures *Altocumulus* and *Cirrocumulus*, respectively. These measurements have been described in our previous publications [13–15]. As when using the programme Cumulus, the measurer moves a toggle to select two thresholds (grey-level values). The first threshold distinguishes the breast from the background and the programme calculates the total area of the breast. The second threshold, known as the pixel density threshold, is based on what the measurer considers to be the dense tissue and the programme calculates the absolute dense area. This is done separately for the three density measures, each according to their different definitions of density, given above.

**Fig. 1:**
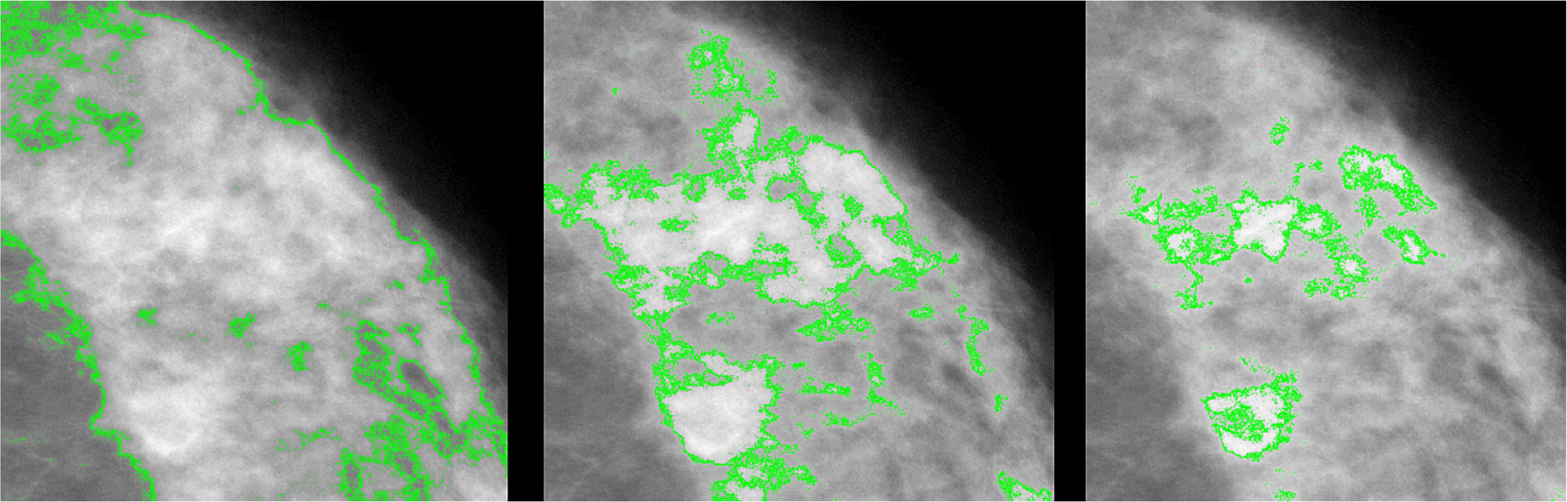
Identification of dense regions (outlined in green) on the same mammogram according to the *Cumulus* (left panel), *Altocumulus* (centre panel) and *Cirrocumulus* (right panel) definitions of mammographic density

Figure 1 shows, for a particular section of the same mammogram, how the dense areas (outlined in green) vary depending on the definition of mammographic density. The left panel shows the region classified as *Cumulus*, and the centre panel shows that within this region there are brighter areas that are classified as *Altocumulus*. Similarly, within the latter regions, the right panel shows that there are even brighter areas that are classified as *Cirrocumulus*.

For each definition of mammographic density, we used the average over all measurers of their transformed, age-adjusted, BMI-adjusted and standardised measures so as to maximise the accuracy; see “Statistical methods”. Correlation between the resulting dense area and percent density measures was 0.87, 0.92, and 0.96 for *Cumulus*, *Altocumulus*, and *Cirrocumulus*, respectively. Correlation between the *Cumulus* and *Altocumulus* measures was 0.86 for dense area and 0.88 for percent density. The corresponding correlation between the *Cumulus* and *Cirrocumulus* measures was 0.82 and 0.82, respectively, and the corresponding correlation between the *Altocumulus* and *Cirrocumulus* measures was 0.82 and 0.82, respectively.

Repeatability was assessed by performing the measurements in 18 sets of 100 mammograms with the same ratio of cases and controls and including 10% repeat samples randomly in each set, to estimate intra-class correlation within the set. In the 5th, 10^th^, and 15th sets, there was also a 10% repeat sample from the first batch to estimate intra-class correlation between the sets. Consequently, 110 images were included in all batches except for batches 5, 10, and 15, which had 120 images.

For dense area, the within-set repeatability was 0.95 (95% confidence interval 0.94–0.96), 0.96 (0.94–0.97) and 0.94 (0.90–0.94) for the average of *Cumulus*, *Altocumulus*, and *Cirrocumulus*, respectively. For dense area, the between-set repeatability was 0.95 (0.93–0.97), 0.95 (0.94–0.96) and 0.94 (0.92–0.94) for the average of *Cumulus*, *Altocumulus*, and *Cirrocumulus*, respectively. Similar repeatability was obtained for the percent density measures. The average of the inter-observer correlation was 0.90, 0.92, and 0.87 for *Cumulus*, *Altocumulus*, and *Cirrocumulus*, respectively.

### Statistical methods

Unconditional logistic regression was used to test for differences between independent sub-groups of cases defined by mode of detection. Conditional logistic regression was used to test for differences between cases and matched controls, and to estimate odds ratios between the mammographic density measures and breast cancer risk, separately for screen-detected breast cancer and interval breast cancer, adjusting for covariates. Given the change in direction of association between BMI and breast cancer risk with increasing age, we included an interaction term to test if the odds ratio for BMI in relation to breast cancer risk depended on age at mammogram. Each mammographic density measure was transformed using a Box–Cox power transformation to have an approximately normal distribution by using maximum likelihood to estimate the parameter λ such that (Y^λ^ – 1)/λ is closest to a normal distribution [20]. For each fitted model, the mean of the transformed measures for the controls was adjusted for age and BMI to derive the residuals, from which their standard deviation was calculated.

As in our previous publications [13–15], we used this adjusted standard deviation and conditional logistic regression to estimate the odds ratio per adjusted standard deviation (OPERA, [21]). OPERA is a measure of the ability of a risk factor (appropriately adjusted for the covariates taken into account by design and analysis) to discriminate between cases and controls. When we fitted two standardised density measures, *X*_1_ and *X*_2_, into the same model, we presented the risk estimates in terms of their standard deviation (SD) after adjusting also for the other measure; see (14). We did this by multiplying the log(OR) estimate from fitting *X*_j_ by SD = [(1 – *r*^2^)]^0.5^, where *r* is the correlation between *X*_1_ and *X*_2_, and then exponentiated it to obtain the appropriate OPERA. The inter-quartile risk ratio (IQQR) corresponding to a given OPERA is approximately OPERA^2.5^. We also used the area under the receiver operator curve (AUC) to measure the discriminatory ability of measures. All statistical analyses were conducted using the software Stata [22], and following convention, *P* < 0.05 was considered to be statistically significant.

Parameter estimates were derived under asymptotic maximum likelihood theory. We compared the fits of nested models using the likelihood ratio criteria; under the null hypothesis, twice the absolute difference in the (maximum) log likelihood (ΔLL) has an approximately χ^2^ distribution with degrees of freedom equal to the difference in number of parameters. The fits of models were also compared based on the differences in the Bayesian information criterion (ΔBIC), which for models with the same number of parameters is 2ΔLL. If ΔBIC is the BIC of model A minus the BIC score of model B, then exp (-ΔBIC) is approximately equal to the posterior odds in favor of model A; for example, ΔBIC = − 6 or − 14 means that model A is roughly 400 or 1,000,000 times, respectively, more likely to be the model that generated the data than model B. The strengths of evidence against the model with the poorest fit are conventionally interpreted as non-existent if < 2, positive if 2–6, strong if 6–10, and very strong if ≥ 10 [23].

## Results

Table 1 shows that women with interval breast cancer did not differ from their matched controls in any baseline characteristic, including BMI, except for family history (*P* ≤ 0.001). Screen-detected cases differed from their matched controls in family history (*P* < 0.001) and BMI (*P* = 0.003).

Table 2 shows that the unadjusted *Cumulus*, *Altocumulus* and *Cirrocumulus* measures of dense area were all greater for cases than for controls, for both interval and screen-detected breast cancer (all *P* < 0.01). Percent dense area measured by *Cumulus*, *Altocumulus* and *Cirrocumulus* was greater for cases with interval breast cancers compared with (1) their controls (all *P* < 0.001), and (2) cases with screen-detected breast cancers (all *P* < 0.001).

**Table 2:** Mammographic density measurements for cases and controls, by mode of detection (screen-detected or interval cancer)

	Interval cancer			Screen-detected cancer		
	Cases (*n* = 168) Mean (SD)	Controls (*n* = 498) Mean (SD)	*P* ^a^	Cases (*n* = 422) Mean (SD)	Controls (*n* = 1197) Mean (SD)	*P* ^a^
*Cumulus*						
Dense area (cm^2^)	25 (13)	19 (11)	< 0.001	21 (13)	19 (12)	0.001
Non-dense area (cm^2^)	110 (54)	126 (56)	0.001	132 (59)	127 (55)	0.2
Percent dense area	21 (11)	15 (9)	< 0.001	16 (10)	14 (9)	0.01
Total area (cm^2^)	135 (56)	145 (56)	0.04	153 (58)	146 (55)	0.04
*Altocumulus*						
Dense area (cm^2^)	12 (8)	9 (7)	< 0.001	10 (7)	9 (8)	0.01
Non-dense area (cm^2^)	124 (57)	137 (57)	0.008	144 (59)	138 (55)	0.09
Percent dense area	11 (8)	7 (6)	< 0.001	7 (6)	7 (6)	0.04
Total area (cm^2^)	136 (57)	146 (57)	0.05	154 (59)	147 (55)	0.04
*Cirrocumulus*						
Dense area (cm^2^)	6.8 (6.2)	4.2 (4.6)	< 0.001	4.8 (5.3)	3.9 (4.9)	0.002
Non-dense area (cm^2^)	129 (55)	141 (56)	0.01	148 (58)	142 (55)	0.09
Percent dense area	6.0 (5)	3.0 (4)	< 0.001	4.0 (4)	3.0 (3)	0.003
Total area (cm^2^)	135 (56)	145 (56)	0.05	153 (58)	146 (55)	0.05

*SD* standard deviation

^a^*P* indicates significance of differences between cases and matched controls based on conditional logistic regression

For interval cancer, the association with BMI did not depend on age at mammogram (*P* > 0.8). There was no association with BMI overall (see Table 1). Screen-detected cancer was associated with BMI and the association became stronger with age at mammogram (*P* < 0.02).

Table 3 shows that, for interval breast cancer, after adjusting for age and BMI all of the dense area measures were associated with breast cancer risk (all *P* < 0.001). The log (OPERA) was 0.61, 0.50 and 0.61 for *Cumulus*, *Altocumulus* and *Cirrocumulus*, respectively (all *P* < 0.001), There was very strong evidence that the *Cumulus* and *Cirrocumulus* measures gave a better fit than the *Altocumulus* measure (both ΔBIC ≥ 10).

**Table 3:** For interval breast cancer, marginal odds ratios, area under the receiver operating characteristic curve (AUC) and log likelihood (LL) for *Cumulus*, *Altocumulus* and *Cirrocumulus* measures of dense area and percent density, adjusted for age and body mass index (BMI)

		Dense area		Percent density
		OR (95% CI)^a^		OR (95% CI)^a^
*Cumulus*	Q1 = 20^b^	–	10	–
	Q2 = 29	1.53 (0.81–2.89)	26	2.51 (1.14–5.52)
	Q3 = 41	2.09 (1.14–3.85)	41	5.11 (2.37–11.1)
	Q4 = 78	4.34 (2.43–7.73)	91	10.4 (4.98–21.8)
	OPERA	1.84 (1.50–2.26)		2.33 (1.85–2.92)
	AUC (95%CI)^c^	0.70 (0.65–0.74)		0.75 (0.70–0.79)
	LL	− 206.95		− 192.48
*Altocumulus*	24	–	18	–
	25	1.06 (0.57–1.98)	22	1.25 (0.62–2.50)
	49	1.82 (1.04–3.18)	48	2.73 (1.46–5.09)
	70	3.09 (1.78–5.36)	80	4.74 (2.57–8.72)
	OPERA	1.65 (1.35–2.00)		1.92 (1.55–2.36)
	AUC (95% CI)^c^	0.67 (0.63–0.72)		0.70 (0.66–0.75)
	LL	− 212.59		− 204.60
*Cirrocumulus*	18	–	15	–
	31	1.47 (0.75–2.88)	29	1.78 (0.90–3.51)
	39	2.33 (1.21–4.49)	40	3.37 (1.71–6.66)
	80	4.64 (2.51–8.58)	84	6.56 (3.44–12.5)
	OPERA	1.84 (1.51–2.25)		2.06 (1.67–2.54)
	AUC (95% CI)^c^	0.70 (0.66–0.75)		0.73 (0.68–0.77)
	LL	− 205.43		− 198.45

Odds ratios (OR) are shown by quartiles (number of cases shown in parentheses)

*OPERA* change in OR per adjusted standard deviation, *CI* confidence interval

^a^Odds ratio per standard deviation adjusted for age and BMI

^b^Quartiles (Q1–Q4) defined by distribution of the measure adjusted for age, BMI and number of livebirths

^c^AUCs for mammographic measurements after adjusting for age and BMI

For percent density measures, there was very strong evidence that the OPERA estimates were all higher than for their corresponding absolute density measure (all ΔBIC ≥ 10), especially for *Cumulus* where log (OPERA) was 0.85 compared with 0.65 and 0.72 for *Altocumulus* and *Cirrocumulus*, respectively (all *P* < 0.001). and that the *Cumulus* measure gave a better fit than the *Cirrocumulus* and *Altocumulus* measures (both ΔBIC ≥ 10). The risk gradient for *Cirrocumulus* was 30% and 17% higher than for the other two measures, respectively. Furthermore, there was very strong evidence that the *Cumulus* percent density measure gave a better fit than the *Cumulus* absolute density measure (ΔBIC = 28), with an estimated risk gradient 39% higher. Univariately, therefore, the *Cumulus* percent density measure gave a better fit for interval cancers, with an estimated risk gradient equivalent to an IQQR ~ 8-fold (95% CI 5–15).

Table 4 shows that, for screen-detected breast cancer, all the dense area measures were associated with risk, after adjusting for age and BMI as an interaction due to the aforementioned findings (paragraph 3, “Results”) (*P* < 0.001). The log OPERA was 0.22, 0.22 and 0.28 for the transformed, normalized and standardized *Cumulus*, *Altocumulus* and *Cirrocumulus* measures, respectively (all *P* < 0.001). There was strong evidence that the *Cirrocumulus* measure gave a better fit than the *Cumulus* and *Altocumulus* measures (ΔBIC = 8). On the log odds scale, the risk gradient for *Cirrocumulus* was 24% higher than for the other two measures. Results were similar for the percent density measures adjusted for age and BMI.

**Table 4:** For screen-detected breast cancer, marginal odds ratios, area under the receiving operating characteristic curve (AUC) and log likelihood (LL) for *Cumulus*, *Altocumulus* and *Cirrocumulus* measures of dense area and percent density adjusted for age and body mass index (BMI)

		Dense area		Percent density
		OR (95% CI)^a^		OR (95% CI)^a^
*Cumulus*	Q1 = 88^b^	–	81	–
	Q2 = 96	1.01 (0.72–1.41)	94	1.20 (0.85–1.69)
	Q3 = 88	0.99 (0.70–1.39)	98	1.12 (0.80–1.59)
	Q4 = 150	1.66 (1.22–2.27)	149	1.95 (1.41–2.70)
	OPERA	1.25 (1.12–1.40)		1.30 (1.16–1.45)
	AUC (95% CI)^c^	0.63 (0.60–0.66)		0.64 (0.61–0.67)
	LL	− 542.27		− 539.87
*Altocumulus*	81	–	85	–
	97	1.19 (0.84–1.66)	85	1.00 (0.71–1.42)
	98	1.28 (0.92–1.79)	114	1.31 (0.95–1.81)
	146	1.86 (1.35–2.56)	138	1.72 (1.24–2.37)
	OPERA	1.25 (1.12–1.40)		1.27 (1.14–1.43)
	AUC (95% CI)^c^	0.63 (0.60–0.66)		0.64 (0.60–0.67)
	LL	− 542.23		− 541.29
*Cirrocumulus*	79	–	72	–
	94	1.32 (0.93–1.87)	408	1.63 (1.15–2.32)
	102	1.38 (0.98–1.94)	99	1.35 (0.95–1.92)
	147	2.08 (1.50–2.87)	143	2.29 (1.63–3.22)
	OPERA	1.32 (1.18–1.48)		1.35 (1.20–1.52)
	AUC (95% CI)^c^	0.64 (0.61–0.67)		0.64 (0.61–0.68)
	LL	−538.21		−536.75

Odds ratios (OR) are shown by quartiles (number of cases shown in parentheses

*OPERA* change in OR per adjusted standard deviation, *CI* confidence interval

^a^OR per standard deviation adjusted for age and BMI

^b^Quartiles (Q1–Q4) defined by distribution of the measure adjusted for age, BMI and number of livebirths

^c^AUCs for mammographic measurements after adjusting for age and BMI

Table 5 shows that, for interval breast cancer, once we adjusted for *Cumulus* as a percentage, using the likelihood ratio test and comparing with the log likelihood of 192.48 from Table 3 showed there was no evidence that adding one or both of the other measures improved the fit (all *P* > 0.1). For example, when fitted together the OPERAs for the *Cumulus* attenuated but remained significant (*P* < 0.001) whereas the *Altocumulus* and *Cirrocumulus* estimates were reduced substantially and were no longer significant (all *P* > 0.07). In particular, adding the *Cirrocumulus* measure made no improvement to the fit (ΔLL = 0.7).

**Table 5:** For interval and screen detected breast cancer, multivariable odds ratios, area under the receiving operating characteristic curve (AUC), and log likelihood (LL) for *Cumulus*, *Altocumulus* and *Cirrocumulus* measures of dense area and percent density adjusted for age and body mass index (BMI)

		Interval cancer	*P*	LL	Screen-detected cancer	*P*	LL
		OPERA (95% CI)			OPERA (95% CI)		
Dense area							
Model 1^a^	*Cumulus*	1.40 (1.15–1.72)	0.001	− 206.91	1.05 (0.94–1.19)	0.3	− 541.75
	*Altocumulus*	0.97 (0.80–1.18)	0.8		1.06 (0.94–1.19)	0.3	
Model 2^b^	*Cumulus*	1.16 (0.94–1.43)	0.2	− 204.46	0.98 (0.87–1.10)	0.8	− 538.16
	*Cirrocumulus*	1.26 (1.02–1.53)	0.03		1.19 (1.06–1.34)	0.005	
Model 3^c^	*Altocumulus*	0.94 (0.76–1.16)	0.6	− 205.27	0.98 (0.87–1.10)	0.8	− 538.18
	*Cirrocumulus*	1.49 (1.20–1.87)	< 0.001		1.19 (1.06–1.34)	0.005	
Model 4^d^	*Cumulus*	1.27 (0.99–1.62)	0.06	− 203.34	0.99 (0.85–1.14)	0.8	− 538.16
	*Altocumulus*	0.84 (0.65–1.07)	0.2		0.99 (0.86–1.15)	0.9	
	*Cirrocumulus*	1.36 (1.07–1.72)	0.01		1.19 (1.04–1.36)	0.008	
Percent density							
Model 1^a^	*Cumulus*	1.67 (1.36–2.07)	< 0.001	− 191.69	1.11 (0.99–1.25)	0.09	− 539.81
	*Altocumulus*	0.88 (0.72–1.07)	0.2		1.02 (0.91–1.15)	0.7	
Model 2^b^	*Cumulus*	1.48 (1.19–1.95)	< 0.001	− 192.00	1.02 (0.91–1.15)	0.7	− 536.68
	*Cirrocumulus*	1.10 (0.90–1.36)	0.3		1.17 (1.03–1.31)	0.01	
Model 3^c^	*Altocumulus*	1.05 (0.85–1.31)	0.6	− 198.33	0.99 (0.89–1.11)	0.9	− 536.74
	*Cirrocumulus*	1.43 (1.16–1.77)	0.001		1.20 (1.06–1.34)	0.003	
Model 4^d^	*Cumulus*	1.68 (1.30–2.19)	< 0.001	− 190.33	1.05 (0.90–1.27)	0.5	− 536.56
	*Altocumulus*	0.79 (0.61–1.02)	0.07		0.96 (0.82–1.12)	0.6	
	*Cirrocumulus*	1.21 (0.96–1.52)	0.1		1.18 (1.04–1.34)	0.01	

The change in odds ratio per adjusted standard deviation (OPERA) adjusted for age and body mass index is shown by quartiles (number of cases shown in parentheses)

CI, confidence interval

^a^*Cumulus* and *Altocumulus* measures fitted together

^b^*Cumulus* and *Cirrocumulus* measures fitted together

^c^*Altocumulus* and *Cirrocumulus* measures fitted together

^d^*Cumulus, Altocumulus* and *Cirrocumulus* measures fitted together

On the other hand, Table 5 shows that for screen-detected breast cancer it was *Cirrocumulus* that was a significant predictor (all *P* < 0.01) irrespective of which other measure(s) were in the model, and for both dense area and percent dense area measures. After adjusting for *Cirrocumulus*, using the likelihood ratio test and comparing with the log likelihoods of − 538.21 and − 536.75 from Table 4, respectively, showed no evidence that adding the other measures improved the fit (all ΔLL < 0.1). In particular, the *Cumulus* measures no longer predicted risk in all multivariable models; their OPERAs were close to 1.0 and not significant whether considered as percent or absolute dense area.

**Fig. 2:**
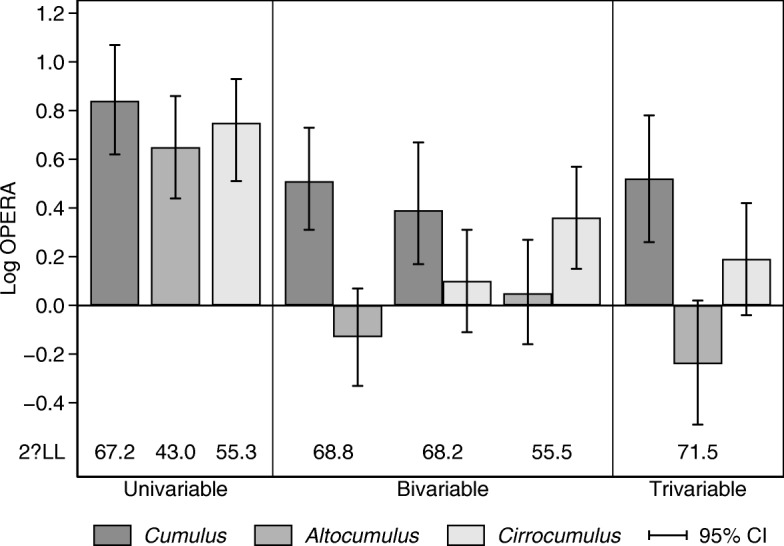
Log odds ratio per adjusted standard deviation (Log OPERA) estimates, 95% confidence intervals (CI) and goodness-of-fit relative to the null model, given by twice the absolute change in log likelihood (2ΔLL) for the univariable, bivariable and trivariable model fits presented in Tables 3 and 5, for interval breast cancer and percent density measures of *Cumulus*, *Altocumulus* and *Cirrocumulus*

**Fig. 3:**
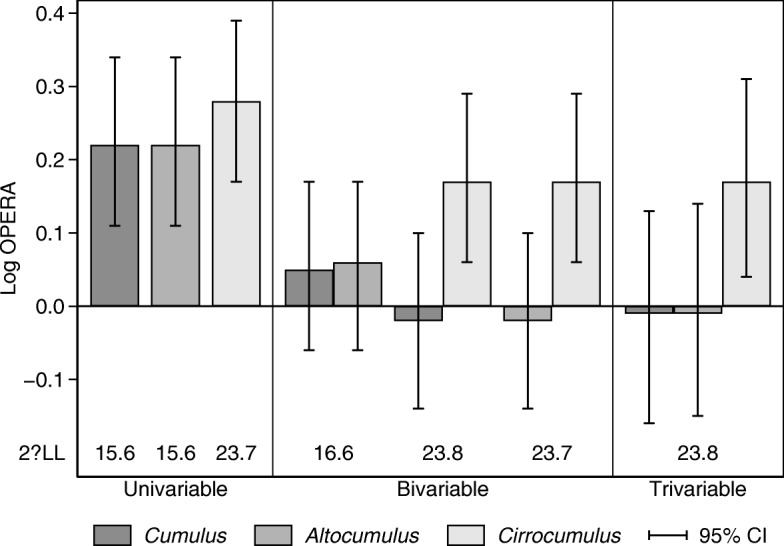
Log odds ratio per adjusted standard deviation (Log OPERA) estimates, 95% confidence intervals (CI) and goodness-of-fit relative to the null model, given by twice the absolute change in log likelihood (2ΔLL) for the univariable, bivariable and trivariable model fits presented in Tables 4 and 5 , for screen-detected breast cancer and density measures of *Cumulus*, *Altocumulus* and *Cirrocumulus*

Figures 2 and 3 illustrate the relative stability of the risk measures by plotting the OPERAs and their confidence intervals as reported in Table 3, 4 and 5, on the log scale. For the percentage measures and interval cancers, the *Cumulus* measure dominates (see Fig. 2), while for dense measures and interval cancers the *Cirrocumulus* measure dominates (see Fig. 3). The patterns in estimates across the models are seen to be clearly different for the two outcomes.

Additional file 1: Tables S1 and S2 show the results of analyses when restricted to invasive cancers only. The aforementioned general patterns observed were maintained.

Additional file 1: Table S3 shows that fitting different models to different modes of detection made a highly significant improvement in fit compared with fitting one model to the combined data - which assumes that estimates are the same for both screen-detected and interval cancers - for all models (all *P* < 0.005).

## Discussion

We found that the risk predictors for breast cancer differ by mode of detection. First, there was very strong evidence that interval breast cancers are best predicted when mammographic density was defined conventionally as the white or bright areas, the more so when defined as a percentage. It was more than 20,000 times more likely to be the measure that generated the data than either of the other two measures. Addition of the other measures did not add information, and BMI did not appear to be a predictor of interval disease. On the other hand, we found very strong evidence that screen-detected breast cancers are best predicted by mammographic density when defined as the brighter areas on a mammogram, whether as an absolute or percentage measure. The *Cirrocumulus* measure was more than 3000 times more likely to be the measure that generated the data than either of the other two measures. Addition of the conventional measures did not add information. Also, in contrast to risk of interval disease, the risk of screen-detected breast cancer associated with BMI increased with age, similar to previous findings on the risk of breast cancer per se [24].

Most importantly, the conventional conception of mammographic density (which we call *Cumulus*) was not predictive of screen-detected cancers once we fitted mammographic density defined at, in effect, a high pixel-brightness threshold. We have observed this before in case-control studies of breast cancer, for both Australian and Korean women, and when using screen-film and digital mammograms [13–15].

Our general observation that risk prediction can be improved by defining density at a higher pixel-brightness threshold has been replicated by Wang and colleagues [25] using digital mammograms. They found, as we have repeatedly found, that once the measure at a higher threshold was included, addition of the conventional measure made no improvement to the fit.

We previously argued that screen-detected cancers mostly reflect inherent risk [12]. We found accumulating evidence that the conventional concept of mammographic density does not apply to risk per se and might be only a surrogate for those aspects of a mammographic image that truly predict risk.

We had also previously noted that interval cancers could be influenced by masking, more rapid growth and/or inherent risk [12]. In this regard, our finding (as previously reported [12]) that the association between BMI and risk of interval cancer did not depend on age, whereas the association between BMI and screen-detected cancer increased with age, is important. The latter is consistent with the epidemiological literature on inherent breast cancer risk (see most recently [26]). This is consistent with risk of interval cancers being more due to masking and more rapid growth than to an increased inherent risk per se and might help explain why we found different mammographic density measures better predicted the different outcomes.

It is not true that, because the three measures are correlated, they are equal proxies of the same underlying exposure. If this was true then the estimates of association would all move towards the null and the width of the confidence intervals would increase substantially when the measures were modelled together. But this did not happen. For screen-detected cancers, when absolute *Cirrocumulus* was fitted with the other two measures, its association remained significant while the other estimates collapsed, and the standard error of its association of the log scale increased by only 17% from 0.058 to 0.068. On the other hand, the standard error for the now null *Cumulus* and *Altocumulus* associations increased by 31% and 30%, respectively. Similar patterns were observed for interval cancer, this time with the *Cumulus* estimate being the stable one. In the previous study of breast cancer and mammographic density using the Melbourne Collaborative Cohort Study [18], a different set of investigators applied the conventional definition to measure mammographic density (correlation between the *Cumulus* measure used in this study and the *Cumulus* measure used in the previous study was 0.86 for dense area and 0.87 for percent dense area). The consistency of our findings on interval cancers with our previous findings based on measurements by other investigators [12], gives confidence in the ability of our investigators performing the measurements in the current study.

The major implication of our findings is that analyses of mammographic images can be used to predict both masking and risk, but that different aspects of the image need to be considered. To date, prediction of masking has been based on BI-RADS, which has in effect been defined in terms of percent density as a categorical variable. Given that we and others [1] have now found that the most informative predictor of masking is a continuous variable, *Cumulus* as a percentage, this raises issues about whether the current thresholds for defining dense breasts for the purposes of recommending other screening modalities are appropriate. Moreover, the implications of having mammographically dense breasts depend on a woman’s underlying risk of breast cancer, and we have shown that the *Cirrocumulus*, rather than the *Cumulus*, measure is an important predictor of risk. Therefore, in order to deal more effectively with the issue of mammographically dense breasts protocols should be devised that take into account that there are multiple and different features of mammographic images that give clinically-important information about different outcomes.

Potential limitations of this study include the older age at baseline of the cohort and, hence, the diagnosis of breast cancer at older ages. It is interesting to note that the OPERA for our predictors of screen-detected cancers was about 1.4, even for *Cirrocumulus*, consistent with the literature on conventional measures of risk [21], whereas we previously found stronger risk discrimination for predicting breast cancers at younger ages for both Australian [13] and Korean [14, 15] women. It is therefore plausible that the risk discrimination (in the sense we have defined it) is weaker the older the age at measurement, and the longer the time interval between measurement and diagnosis (despite the very strong tracking in age-adjusted mammographic density risk measures over time [27]).

Another limitation is that this study used screen-film images, whereas digital mammography is now the norm. Our previous case–control studies have shown that similar findings on risk arise from studies of digital and film mammograms, but it is nevertheless important to replicate our findings using digital mammography, and for diagnoses at different ages and with different time intervals in different populations. This requires new prospective studies, and we and others are working on developing such resources. Another limitation of this study is that we were not able to divide the period of 2 years used to define interval breast cancers occurring into shorter time periods, such as 12 months as in Boyd et al. [1].

A potential limitation of the translation of our findings into clinical settings is that the measurements require person time and expertise. With respect to expertise, the investigators who performed the measurements are not radiologists but have achieved high repeatability within and between measurers, as aforementioned. We have been funded to train radiologists using the online, and therefore globally relevant, BreastScreen Reader Assessment Strategy initiative [28]. We are also working with colleagues on developing automated measures.

One strength of this study is the highly statistically significant and consistent results observed despite the fact that multiple measurers have been used and the definitions of the different density measurements involve subjective judgements. This is testament to the high repeatability of the average of risk measures across our trained measurers. It also suggests that there is the potential to derive even better predictors of risk, as random measurement error contributes to underestimation of risk gradients. For example, the measurers were blinded to their previous mammographic density measurements on the same mammogram, and better risk prediction might be possible if *Cumulus*, *Cirrocumulus* and *Altocumulus* were measured in that order at the same time, un-blinded to the previous measure. Automated methods would allow multiple thresholds to be considered and, thus, could determine an optimal risk-predicting threshold.

Another strength has been our use of the OPERA concept, which allows us to put risk factors into perspective in terms of their ability to differentiate cases from controls. OPERA relies on deriving the standard deviation of the risk factor after adjusting for other covariates and design variables. Not taking into account the need to adjust the standard deviation for covariates can make a substantial difference. For example, for unadjusted transformed percent mammographic density, the cross-sectional standard deviation was 0.63 for controls. However, after adjusting for age and body mass index, the standard deviation of the residuals became 0.34. Consequently, the log (OPERA) was 44% less than the log (OR) per cross-sectional standard deviation; not adjusting the standard deviation leads to an almost 50% overestimate of the risk-predicting ability of percent mammographic density.

## Conclusion

In summary, we have found that more risk-predicting information can be obtained from a mammogram than from the conventional concept of mammographic, or breast, density defined by the white or bright areas. We have also confirmed that the conventional measure of percent mammographic density applies to interval cancers and the masking phenomenon. When it comes to risk per se, however, we have confirmed our previous findings that the conventional measures are not the most informative mammography-based predictors, given that they are no longer significant after adjustment for our new measures based on higher pixel-brightness thresholds. Our newer measures could also be clinically useful given that we have shown that non-specialists can be easily trained and achieve high repeatability within and between measurers. For scientific accuracy, we have used the average of five measurements by five investigators, but reasonable accuracy was obtained by each individual investigator (data not shown); see Fig. 2 of [15] which shows an example of measures of the same mammograms conducted by different investigators.

Our finding that areas of high pixel brightness might better represent the breast tissue with a causal role in breast cancer is very important for multiple disciplines, such as biological research to identify specific tissue markers [29], genetic research to try to find pathways implicated in disease risk [30] and public health initiatives aimed towards targeted, or tailored screening, by identifying women at increased risk of masking and/or of the disease itself.

## Additional file

Additional file 1: **Table S1.** For interval and screen-detected invasive breast cancer, marginal risk estimates for *Cumulus*, *Altocumulus* and *Cirrocumulus* measures of dense area and percent density adjusted for age and BMI. **Table S2.** For interval and screen-detected invasive breast cancer, multivariable risk estimates for *Cumulus*, *Altocumulus* and *Cirrocumulus* measures of dense area and percent density adjusted for age and BMI. **Table S3.** Combined (all breast cancer); total (summary of interval and screen-detected); and -2Δ of log likelihood (LL) for *Cumulus*, *Altocumulus* and *Cirrocumulus* measures of dense area and percent density adjusted for age and BMI and *P* value for the differences. (DOCX 36 kb)

## Abbreviations

AUC: Area under the receiver operating characteristic curve
BIC: Bayesian information criterion
BMI: Body mass index
CC: Cranio-caudal
CI: Confidence interval
HRT: Hormone replacement therapy
LL: Log likelihood
OPERA: Odds ratio per adjusted standard deviation
