# Supplementary material for: Predicting interval and screen-detected breast cancers from mammographic density defined by different brightness thresholds
Source: Breast Cancer Res. 2018 Dec 13;20:152. doi: 10.1186/s13058-018-1081-0 (PMC6293866; doi:10.1186/s13058-018-1081-0)
Supplement: Supplementary file 1 — Table S1. For interval and screen-detected invasive breast cancer, marginal risk estimates for Cumulus, Altocumulus and Cirrocumulus measures of dense area and percent density adjusted for age and BMI. Table S2. For interval and screen-detected invasive breast cancer, multivariable risk estimates for Cumulus, Altocumulus and Cirrocumulus measures of dense area and percent density adjusted for age and BMI. Table S3. Combined (all breast cancer); total (summary of interval and screen-detected); and -2Δ of log likelihood (LL) for Cumulus, Altocumulus and Cirrocumulus measures of dense area and percent density adjusted for age and BMI and P value for the differences. (DOCX 36 kb) [file 13058_2018_1081_MOESM1_ESM.docx]

**Table S1.** For Interval and screen-detected invasive breast cancer, marginal risk estimates for *Cumulus*, *Altocumulus* and *Cirrocumulus* measures of dense area and percent density adjusted for age and BMI.

|  |  | **Dense Area** | |  | **Percent Density** | |
| --- | --- | --- | --- | --- | --- | --- |
| **Interval** | | **OR (95% CI)^a^** | **AUC (95%CI)^b^** |  | **OR (95% CI)^a^** | **AUC (95%CI)^b^** |
| *Cumulus* | |  |  |  |  |  |
|  | OPERA | 1.93 (1.54–2.42) | 0.70 (0.65–0.74) |  | 2.43 (1.90–3.11) | 0.75 (0.70–0.79) |
| *Altocumulus* | |  |  |  |  |  |
|  | OPERA | 1.74 (1.41–2.15) | 0.68 (0.63–0.72) |  | 2.00 (1.60–2.50) | 0.70 (0.66–0.75) |
| *Cirrocumulus* | |  |  |  |  |  |
|  | OPERA | 1.90 (1.53–2.37) | 0.70 (0.66–0.75) |  | 2.13 (1.69–2.68) | 0.73 (0.69–0.77) |
|  |  |  |  |  |  |  |
| **Screen-detected** | | **OR (95% CI)^a^** | **AUC (95%CI)^b^** |  | **OR (95% CI)^a^** | **AUC (95%CI)^b^** |
| *Cumulus* | |  |  |  |  |  |
|  | OPERA | 1.15 (1.02–1.30) | 0.62 (0.58–0.65) |  | 1.20 (1.06–1.36) | 0.62 (0.59–0.66) |
| *Altocumulus* | |  |  |  |  |  |
|  | OPERA | 1.17 (1.03–1.32) | 0.62 (0.58–0.65) |  | 1.19 (1.06–1.35) | 0.62 (0.59–0.66) |
| *Cirrocumulus* | |  |  |  |  |  |
|  | OPERA | 1.21 (1.07–1.37) | 0.62 (0.59–0.66) |  | 1.25 (1.10–1.41) | 0.63 (0.59–0.66) |

^a^ OR, odds ratio per standard deviation adjusted for age and body mass index; CI, confidence interval;

^b^ AUCs refer to the area under the receiver operating characteristic curves for mammographic measurements after adjusting for age and body mass index.

**Table S2.** For interval and screen-detected invasive breast cancer, multivariable risk estimates for *Cumulus*, *Altocumulus* and *Cirrocumulus* measures of dense area and percent density adjusted for age and BMI.

|  |  |  |  | **Interval cancer** |  |  | **Screen-detected** |  |
| --- | --- | --- | --- | --- | --- | --- | --- | --- |
| **Dense area** | |  |  | **OPERA (95% CI)** | ***P*** |  | **OPERA (95% CI)** | ***P*** |
| Model 1^a^ | | *Cumulus* |  | 1.39 (1.19–1.73) | 0.003 |  | 1.02 (0.90–1.15) | 0.8 |
|  |  | *Altocumulus* | | 1.01 (0.82–1.25) | 0.9 |  | 1.06 (0.94–1.20) | 0.3 |
|  |  |  |  |  |  |  |  |  |
| Model 2^b^ | | *Cumulus* |  | 1.21 (0.94–1.53) | 0.1 |  | 0.97 (0.86–1.10) | 0.6 |
|  |  | *Cirrocumulus* | | 1.23 (0.99–1.54) | 0.07 |  | 1.15 (1.01–1.30) | 0.04 |
|  |  |  |  |  |  |  |  |  |
| Model 3^c^ | | *Altocumulus* | | 1.02 (0.82–1.42) | 0.8 |  | 0.99 (0.87–1.13) | 0.9 |
|  |  | *Cirrocumulus* | | 1.42 (1.13–1.78) | 0.003 |  | 1.13 (0.99–1.27) | 0.07 |
|  |  |  |  |  |  |  |  |  |
| Model 4^d^ | | *Cumulus* |  | 1.27 (0.97–1.67) | 0.08 |  | 0.96 (0.82–1.12) | 0.6 |
|  |  | *Altocumulus* | | 0.91 (0.70–1.18) | 0.5 |  | 1.02 (0.87–1.19) | 0.8 |
|  |  | *Cirrocumulus* | | 1.28 (1.00–1.58) | 0.05 |  | 1.14 (0.99–1.31) | 0.06 |
| **Percent density** | | |  |  |  |  |  |  |
| Model 1^a^ | | *Cumulus* |  | 1.66 (1.33–2.08) | <0.001 |  | 1.06 (0.93–1.21) | 0.4 |
|  |  | *Altocumulus* | | 0.91 (0.74–1.13) | 0.5 |  | 1.03 (0.91–1.18) | 0.6 |
|  |  |  |  |  |  |  |  |  |
| Model 2^b^ | | *Cumulus* |  | 1.55 (1.22–1.97) | <0.001 |  | 1.01 (0.89–1.15) | 0.9 |
|  |  | *Cirrocumulus* | | 1.08 (0.86–1.35) | 0.3 |  | 1.12 (0.99–1.27) | 0.08 |
|  |  |  |  |  |  |  |  |  |
| Model 3^c^ | | *Altocumulus* | | 1.13 (0.90–1.41) | 0.3 |  | 1.01 (0.89–1.14) | 0.9 |
|  |  | *Cirrocumulus* | | 1.39 (1.11–1.75) | 0.005 |  | 1.13 (0.99–1.28) | 0.06 |
|  |  |  |  |  |  |  |  |  |
| Model 4^d^ | | *Cumulus* |  | 1.71 (1.28–2.28) | <0.001 |  | 1.02 (0.86–1.20) | 0.9 |
|  |  | *Altocumulus* | | 0.84 (0.64–1.11) | 0.2 |  | 0.99 (0.85–1.17) | 0.96 |
|  |  | *Cirrocumulus* | | 1.15 (0.89–1.48) | 0.3 |  | 1.13 (0.98–1.29) | 0.09 |

^a^ *Cumulus* and *Altocumulus* measures fitted together;

^b^ *Cumulus* and *Cirrocumulus* measures fitted together;

^c^ *Altocumulus* and *Cirrocumulus* measures fitted together;

^d^ *Cumulus, Altocumulus* and *Cirrocumulus* measures fitted together.

**Table S3:** Combine (all breast cancer); total (summary of interval and screen-detected); and −2*Δ of log likelihood (LL) for *Cumulus*, *Altocumulus* and *Cirrocumulus* measures of dense area and percent density adjusted for age and BMI and *P*_value_ of the differences.

|  |  |  |  | **Combine** | **Total** | **−2*Δ** | ***P*** |
| --- | --- | --- | --- | --- | --- | --- | --- |
|  |  |  |  | **LL** ^e^ | **LL** ^e^ | **LL** ^e^ |  |
| Dense area | |  |  |  |  |  |  |
|  |  | *Cumulus* |  | -754.72 | -749.22 | 11.00 | 0.0009 |
|  |  | *Altocumulus* |  | -757.71 | -754.82 | 5.78 | 0.02 |
|  |  | *Cirrocumulus* |  | -747.79 | -743.64 | 8.30 | 0.004 |
| Percent density | |  |  |  |  |  |  |
|  |  | *Cumulus* |  | -743.57 | -732.35 | 22.44 | 0.000002 |
|  |  | *Altocumulus* |  | -751.83 | -745.89 | 11.88 | 0.0006 |
|  |  | *Cirrocumulus* |  | -741.46 | -735.20 | 12.52 | 0.0004 |
| Dense area | |  |  |  |  |  |  |
|  | Model 1^a^ | *Cumulus* |  | -754.48 | -748.66 | 11.64 | 0.003 |
|  |  | *Altocumulus* |  |  |  |  |  |
|  |  |  |  |  |  |  |  |
|  | Model 2^b^ | *Cumulus* |  | -747.73 | -742.62 | 10.22 | 0.006 |
|  |  | *Cirrocumulus* |  |  |  |  |  |
|  |  |  |  |  |  |  |  |
|  | Model 3^c^ | *Altocumulus* |  | -747.66 | -743.45 | 8.42 | 0.01 |
|  |  | *Cirrocumulus* |  |  |  |  |  |
|  |  |  |  |  |  |  |  |
|  | Model 4^d^ | *Cumulus* |  | -747.36 | -741.50 | 11.72 | 0.008 |
|  |  | *Altocumulus* |  |  |  |  |  |
|  |  | *Cirrocumulus* |  |  |  |  |  |
| Percent density | |  |  |  |  |  |  |
|  | Model 1^a^ | *Cumulus* |  | -743.46 | -731.50 | 23.92 | 0.000006 |
|  |  | *Altocumulus* |  |  |  |  |  |
|  |  |  |  |  |  |  |  |
|  | Model 2^b^ | *Cumulus* |  | -739.47 | -728.68 | 21.58 | 0.00002 |
|  |  | *Cirrocumulus* |  |  |  |  |  |
|  |  |  |  |  |  |  |  |
|  | Model 3^c^ | *Altocumulus* |  | -741.45 | -735.07 | 12.76 | 0.002 |
|  |  | *Cirrocumulus* |  |  |  |  |  |
|  |  |  |  |  |  |  |  |
|  | Model 4^d^ | *Cumulus* |  | -738.26 | -726.89 | 22.74 | 0.00005 |
|  |  | *Altocumulus* |  |  |  |  |  |
|  |  | *Cirrocumulus* |  |  |  |  |  |

^a^ *Cumulus* and *Altocumulus* measures fitted together;

^b^ *Cumulus* and *Cirrocumulus* measures fitted together;

^c^ *Altocumulus* and *Cirrocumulus* measures fitted together;

^d^ *Cumulus, Altocumulus* and *Cirrocumulus* measures fitted together.

^e^ LL: log likelihood
